# What do we know about the epidemiology of avoidant/restrictive food intake disorder in children and adolescents? A systematic review of the literature

**DOI:** 10.1002/erv.2964

**Published:** 2022-12-16

**Authors:** Javier Sanchez‐Cerezo, Lidushi Nagularaj, Julia Gledhill, Dasha Nicholls

**Affiliations:** ^1^ Division of Psychiatry Department of Brain Sciences Imperial College London London UK; ^2^ Research Department of Clinical, Educational and Health Psychology University College London London UK

**Keywords:** ARFID, children and adolescents, epidemiology, incidence, prevalence

## Abstract

**Background:**

Avoidant/restrictive food intake disorder (ARFID) was a new diagnosis in DSM‐5. This systematic review explores what is known to date about the epidemiology of ARFID in children and adolescents.

**Method:**

Embase, Medline and PsycInfo were used to identify studies meeting inclusion criteria. PRISMA guidelines were followed.

**Results:**

Thirty studies met inclusion criteria, with most coming from specialised eating disorder services where prevalence rates were 5%–22.5%. Three studies from specialist feeding clinics showed the highest prevalence rates, ranging from 32% to 64%. Studies from non‐clinical samples reported ARFID prevalence estimates ranging from 0.3% to 15.5%. One study, using national surveillance methodology, reported the incidence of ARFID in children and adolescents reaching clinical care to be 2.02 per 100,000 patients. Psychiatric comorbidity was common, especially anxiety disorders (9.1%–72%) and autism spectrum disorder (8.2%–54.75%).

**Conclusion:**

The current literature on the epidemiology of ARFID in children and adolescents is limited. Studies are heterogeneous with regard to setting and sample characteristics, with a wide range of prevalence estimates. Further studies, especially using surveillance methodology, will help to better understand the nature of this disorder and estimate clinical service needs.

## Highlights

- ARFID prevalence estimates vary widely across studies and are highest in specialist feeding clinics. The one incidence study to date suggests that new presentations to clinical care are relatively rare.
- ARFID has a high rate of psychiatric comorbidity especially with anxiety disorders.
- Further epidemiological studies, especially using national surveillance methodology, will help planning and resource allocation for this patient group.

**Abbreviations**
AN: Anorexia nervosa
ARFID: Avoidant/restrictive food intake disorder
ARFID‐BS: ARFID‐Brief Screener
ASD: Autism spectrum disorder
AXIS: Appraisal tool for Cross‐Sectional Studies
BMI: Body mass index
BN: Bulimia nervosa
CAPSS: Child and Adolescent Psychiatry Surveillance System
DSM‐5: Diagnostic and Statistical Manual of Mental Disorders (Fifth edition)
DSM‐IV: Diagnostic and Statistical Manual of Mental Disorders (Fourth edition)
ED: Eating disorder
EDNOS: Eating disorder not otherwise specified
EDY‐Q: Eating Disturbances in Youth‐Questionnaire
FAED: Food avoidance emotional disorder
GI: Gastrointestinal
GOS: Great Ormond Street
GSD: Glycogen storage disease
ICD‐11: International Classification of Diseases (Eleventh edition)
IKH: Idiopathic ketotic hypoglycaemia
JECS: Japan Environment and Children's Study
K‐SADS‐E: Kiddie Schedule for Affective Disorders and Schizophrenia‐Epidemiological version
LCA: Latent class analysis
NDD: Neurodevelopmental disorder
NDP: Neurodevelopmental problem
NIAS: Nine‐Item ARFID Screen
PARDI: Pica, ARFID, and Rumination Disorder Interview
PSU: Paediatric Surveillance Unit
SE: Selective eating
SPARKS: Simons Foundation Powering Autism Research
UK: United Kingdom
USA: United States of America

## 1 INTRODUCTION

Avoidant/Restrictive Food Intake Disorder (ARFID) is characterised by a persistent disturbance in feeding or eating which results in an inability to meet nutritional and/or energy needs and leads to at least one of the following: weight loss or failure to achieve appropriate weight gain; nutritional deficiency; dependence on enteral feeding or nutritional supplements; or significant interference with psychosocial functioning. ARFID was first included as a specific diagnosis in the Diagnostic and Statistical Manual of Mental Disorders (5th ed.; DSM–5) in 2013 and is now also part of the International Classification of Diseases (11^th^ ed.; ICD‐11) where it replaced ‘Feeding Disorder of Infancy and Early Childhood’, recognising that ARFID symptoms can occur across the lifespan (American Psychiatric Association & DSM‐5 Task Force, 2013; World Health Organization, 2019). ARFID encompasses several terms previously used to describe restrictive eating patterns presenting to clinical services but not meeting criteria for an eating disorder. This classification is more useful clinically and provides a diagnosis for those previously excluded from other feeding or eating diagnoses (Kreipe & Palomaki, 2012). According to the DSM‐5, food restriction in ARFID may be due to high sensitivity to sensory aspects of food (such as texture, colour, or temperature); to a lack of interest in food or eating; or to anxiety around food, including fear of aversive consequences (i.e., choking or vomiting) associated with eating. These three presentations are not mutually exclusive, and an individual can present with one, two, or even all three (American Psychiatric Association & DSM‐5 Task Force, 2013; Brigham et al., 2018; Thomas et al., 2017). Unlike patients with anorexia nervosa, ARFID is not associated with concerns about gaining weight nor with a preoccupation about body weight, shape, or size (American Psychiatric Association & DSM‐5 Task Force, 2013). If the eating disturbance occurs in the context of another condition or disorder, the severity of the eating disturbance must exceed that routinely associated with the condition or disorder and require additional clinical attention (American Psychiatric Association & DSM‐5 Task Force, 2013; Katzman et al., 2019).

Restrictive eating behaviours are common during childhood. Thus, it is important to differentiate clinically relevant food avoidance from typically developing eating behaviours as, for example, most children go through a phase of food neophobia (refusal to accept new food in the habitual diet) but this is usually transient (Dovey et al., 2008). In addition, a feeding difficulty known as picky eating and defined as the consumption of an inadequate variety or quantity of foods through the rejection of both familiar and unfamiliar foods, is common in young people, with a reported prevalence ranging from 5.6% to 59% (Taylor et al., 2015), although children typically expand their diets as they grow older. However, without an intervention the diet of children with ARFID may remain restricted into adulthood (Thomas et al., 2017) and patients are at risk of medical complications due to low weight such as bradycardia, prolonged QT interval, electrolyte abnormalities, lower bone mineral density and amenorrhoea (Alberts et al., 2020; Katzman et al., 2019) as well as complications of vitamin and other micronutrient deficiencies such as scurvy (Sharp et al., 2020) or loss of vision (Chiarello et al., 2018).

ARFID is a heterogeneous entity which includes different clinical presentations with likely multiple aetiologies (Mairs & Nicholls, 2016). To date, little is known about the possible causes of this disorder, although neurodevelopmental disorders, particularly autism spectrum disorder (ASD), may underlie some presentations (Mairs & Nicholls, 2016). The sensory sensitivities that are commonly seen in young people with ASD may predispose to the development of ARFID (Coglan & Otasowie, 2019). In other cases, ARFID may arise in individuals who experienced a traumatic event related to eating, such as choking or abdominal pain, and then become fearful of eating similar foods and avoid their consumption (Coglan & Otasowie, 2019). A biological three‐dimensional approach has been proposed to explain the three subtypes of ARFID with abnormalities in taste perception, activation of the brain's appetite‐regulating centres or fear responsiveness underlying the three ARFID presentations of sensory sensitivity, lack of interest in food or fear of aversive consequences of eating respectively (Thomas et al., 2017).

As a relatively recent diagnosis, there have been limited large scale epidemiological studies on ARFID with its incidence and prevalence yet to be clarified in both general and clinical populations. Estimates of the prevalence of ARFID in children and adolescents vary widely between studies depending on the age of study participants, geographical location of the study and methodological differences. Research to date has shown that ARFID is present in many countries and more commonly seen in clinical settings (Micali et al., 2020). Whilst the vast majority of research on the epidemiology of ARFID has focussed on child and adolescent populations, a number of studies with adult samples have been published in recent years. Estimates of the prevalence of ARFID using population‐based surveys or screening tools ranged from 0.3% to 4.8% in general adult populations across different countries from Oceania, North America, South‐East Asia, or Europe (Fitzsimmons‐Craft, Balantekin, Graham, Smolar, Park, Mysko, Funk, Taylor, Wilfley; Chua et al., 2021; Hilbert et al., 2021; Chua et al., 2022; Hay et al., 2017). In clinical populations, a retrospective chart review of Japanese women with feeding and eating disorders aged 15–40 years revealed that 11% of the sample met DSM‐5 criteria for ARFID (Nakai et al., 2017).

It is important to have greater clarity about the epidemiology of ARFID in different populations. This information may help to inform screening and diagnosis in clinical contexts, increase the awareness of this diagnostic group and influence service planning and resource allocation, as well as the development of evidence‐based interventions. Children and adolescents have distinct clinical needs and access different health care systems from adults. Knowledge of the epidemiology is the best basis for planning treatment as it helps to clarify the number of people in the need of care and consequently the resources required to treat this patient group. However, to our knowledge there are no systematic reviews to date on the prevalence and incidence of ARFID. This work aims to address this gap by reviewing the current literature on the epidemiology of ARFID, focussing on children and adolescents within both clinical and community samples.

## 2 METHODS

### 2.1 Literature search

The protocol was developed in line with the Preferred Reporting Items for Systematic Reviews and Meta‐Analyses (PRISMA) guidelines and prospectively registered on PROSPERO (CRD42021231901).

An initial literature search was carried out in February 2021 using three data bases (Embase, Medline and PsycINFO) using the OVID interface.

Articles published between January 2013 and February 2021 were identified. ARFID was first included in the DSM‐5 as a diagnosis, which was published in 2013. Hence, 2013 was taken as the earliest date of the search.

The following search terms were used: “ARFID” OR “avoidant restrictive food intake disorder”. Search terms were truncated where appropriate and Medical Subject Headings (or equivalents) were used.

Bibliographies of articles were also searched together with the grey literature and experts in the field were consulted to ensure that any additional relevant articles were included.

The search was repeated in October 2022 to identify any further articles since the initial search.

### 2.2 Eligibility criteria

Inclusion criteria: Observational studies published in English which focussed on the epidemiology of ARFID in children and adolescents with an ARFID diagnosis made according to DSM‐5 criteria.

Exclusion criteria: Studies focussing on adults and conference abstracts were excluded.

### 2.3 Data extraction

The titles of the identified papers were initially screened for eligibility. Abstracts of remaining studies were then screened. This was independently carried out by two authors (JSC and LN). Any inconsistencies were resolved through discussion with a third author (JG). Remaining papers were read in full by JSC and LN and data extracted using a MS word form developed for this review.

All articles were included in the EndNote 20 bibliographic reference management programme.

### 2.4 Quality assessment

The methodological quality of the studies was assessed with the Appraisal tool for Cross‐Sectional Studies (AXIS) (Downes et al., 2016). This has 20 questions which can each be answered with “yes”, “no” or “not known”. One point was assigned for each positive response and a total score was derived.

Two authors (JS and LN) performed the quality assessment separately and disagreements were resolved by consensus or through involvement of a third reviewer (JG) when needed.

## 3 RESULTS

### 3.1 Study characteristics

**FIGURE 1:**
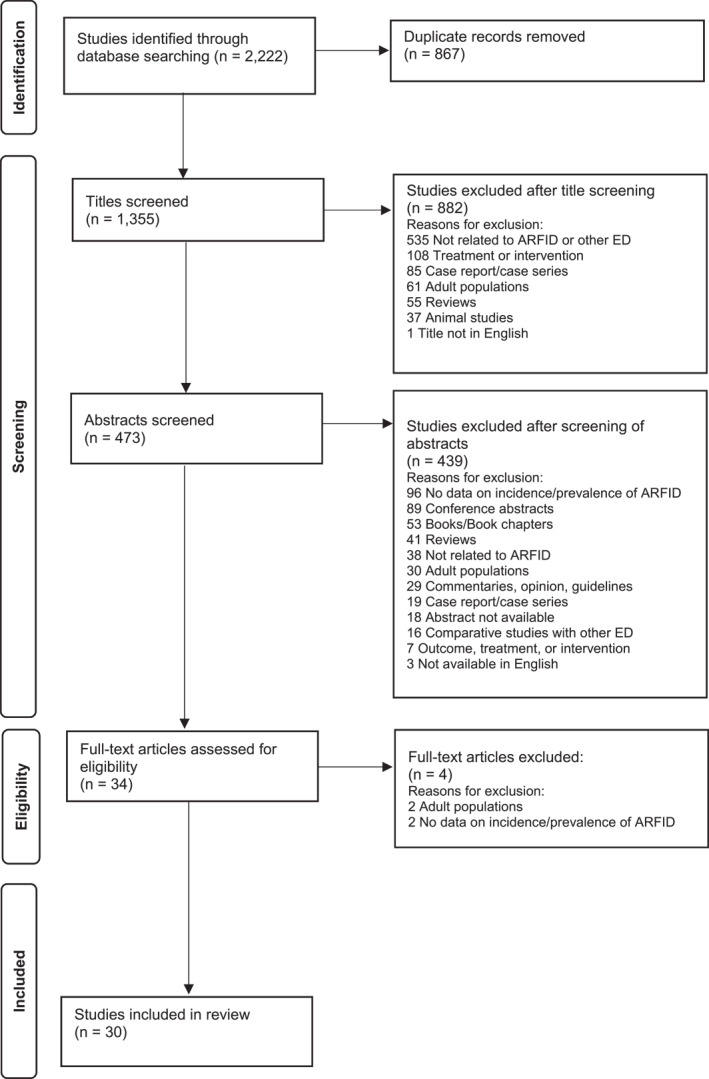
PRISMA flowchart

The PRISMA flowchart is shown in Figure 1 and summarises the stages of the review. Thirty studies met inclusion criteria and were included in the review (Bertrand et al., 2021; Chen et al., 2020; Cooney et al., 2018; Dinkler et al., 2022a, 2022b; Eddy et al., 2015; Farag et al., 2022; Fisher et al., 2014, 2015; Forman et al., 2014; Goldberg et al., 2020; Gonçalves et al., 2019; Iron‐Segev et al., 2022; Katzman et al., 2021; Koomar et al., 2021; Krom et al., 2019; Kurz et al., 2015; Murray et al., 2022; Nicely et al., 2014; Norris et al., 2014; Nygren et al., 2021; Ornstein et al., 2013; Pinhas et al., 2017; Schmidt et al., 2018; Schöffel et al., 2021; Seike et al., 2016a, 2016b; Venema et al., 2022; Williams et al., 2015; Wong et al., 2022). Key aspects of the included studies using clinical samples are presented in Table 1 and those using community and school samples in Table 2. The majority were conducted in Western countries (*n* = 23) (Bertrand et al., 2021; Cooney et al., 2018; Eddy et al., 2015; Farag et al., 2022; Fisher et al., 2014, 2015; Forman et al., 2014; Goldberg et al., 2020; Gonçalves et al., 2019; Katzman et al., 2021; Koomar et al., 2021; Krom et al., 2019; Kurz et al., 2015; Murray et al., 2022; Nicely et al., 2014; Norris et al., 2014; Nygren et al., 2021; Ornstein et al., 2013; Pinhas et al., 2017; Schmidt et al., 2018; Schöffel et al., 2021; Venema et al., 2022; Williams et al., 2015), predominantly the USA (*n* = 9) (Eddy et al., 2015; Fisher et al., 2014, 2015; Forman et al., 2014; Koomar et al., 2021; Murray et al., 2022; Nicely et al., 2014; Ornstein et al., 2013; Williams et al., 2015), with two carried out in multiple countries (Fisher et al., 2014; Pinhas et al., 2017). One study took place in the Middle East (Iron‐Segev et al., 2022) and six in East Asian countries: four in Japan (Dinkler et al., 2022a, 2022b; Seike et al., 2016a, 2016b), one in Taiwan (Chen et al., 2020) and one in Singapore (Wong et al., 2022). The majority (*n* = 21) of studies focussed on clinical populations (Bertrand et al., 2021; Cooney et al., 2018; Eddy et al., 2015; Farag et al., 2022; Fisher et al., 2014, 2015; Forman et al., 2014; Goldberg et al., 2020; Katzman et al., 2021; Koomar et al., 2021; Krom et al., 2019; Murray et al., 2022; Nicely et al., 2014; Norris et al., 2014; Nygren et al., 2021; Ornstein et al., 2013; Pinhas et al., 2017; Schöffel et al., 2021; Venema et al., 2022; Williams et al., 2015; Wong et al., 2022) with many drawing on samples from adolescent eating disorders services or feeding clinics (*n* = 11) (Cooney et al., 2018; Farag et al., 2022; Fisher et al., 2014, 2015; Forman et al., 2014; Krom et al., 2019; Nicely et al., 2014; Norris et al., 2014; Ornstein et al., 2013; Williams et al., 2015; Wong et al., 2022) and utilising retrospective chart review methodology (*n* = 13) (Cooney et al., 2018; Eddy et al., 2015; Fisher et al., 2014; Krom et al., 2019; Murray et al., 2022; Nicely et al., 2014; Norris et al., 2014; Nygren et al., 2021; Ornstein et al., 2013; Schöffel et al., 2021; Venema et al., 2022; Williams et al., 2015; Wong et al., 2022). All studies were cross‐sectional in design and ARFID diagnosis used the DSM‐5 or instruments that were based on the DSM‐5.

**TABLE 1:** Included studies using clinical samples

Author (year)	Country	Design	Methodology	Sample description	Sample setting	Sample size (*n* = )	Sample age	Sample gender	How was the ARFID diagnosis made?	ARFID prevalence/incidence	Other findings
Ornstein et al. (2013)	USA	Cross‐sectional	Retrospective chart review	New patients who presented for initial evaluation to adolescent medicine physicians from six institutions in the US in 2010 or 2011	Adolescent Medicine‐based ED programs	215	8–21 years (15.4 ± 3.3)	88.6% female	The diagnosis was assigned using a diagnostic checklist based on the proposed DSM‐5 diagnostic criteria (either concurrently or retrospectively)	14% (*n* = 31) of participants met ARFID criteria	30 out of 31 patients with ARFID, had a diagnosis of EDNOS according to DSM‐IV
Fisher et al. (2014)	USA and Canada	Cross‐sectional	Retrospective chart review	New patients who presented to seven adolescent medicine eating disorder programs in the US and Canada between January and December 2010	AdolescentMedicine‐based ED programs	712	8–18 years (ARFID age = 12.9 ± 2.5)		The diagnosis was assigned using a diagnostic checklist based on the proposed DSM‐5 diagnostic criteria	13.8% of participants met ARFID criteria	ARFID patients were younger, more likely to be male, had a longer duration of illness, more likely to have a medical condition or an anxiety disorder than those with AN or BN
Norris et al. (2014)	Canada	Cross‐sectional	Retrospective chart review of patients diagnosed with FAED, childhood AN, SE, EDNOS, EDNOS restrictive subtype, or those that were discharged without diagnosis	Patients who received an ED initial assessment between 2000 and 2011	Paediatric ED treatment programme	699	ARFID age = 13.7 ± 2.5 years		DSM‐5 criteria	≈5% of participants met ARFID criteria	Patients with ARFID were younger than those with AN, more likely to present before age 12, and to be male. 12% of ARFID patients' diagnoses were changed to AN restricting subtype over time
Forman et al. (2014)	USA	Cross‐sectional	Retrospective chart review at intake and 1 year follow‐up	Patients who presented restrictive EDs to 14 adolescent medicine based ED programs in calendar year 2010	AdolescentMedicine‐based ED programs	700	9–21 years (15.3 ± 2.4; ARFID age = 13.8 ± 2.6)	86.3% female	The diagnosis was assigned using a diagnostic checklist based on the proposed DSM‐5 diagnostic criteria	12.4% of participants met ARFID criteria	ARFID were more likely to be male, younger, and had a longer duration of illness than AN
Nicely et al. (2014)	USA	Cross‐sectional	Retrospective chart review	Patients admitted to a day programme for children and adolescents with EDs between August 2008 and May 2012	Day programme for ED	173	7–17 years (13.5 ± 2.03; ARFID age = 11.1 ± 1.7)	92% female	The diagnosis was assigned using a diagnostic checklist based on the proposed DSM‐5 diagnostic criteria	22.5% of participants met ARFID criteria	The ARFID group was younger and had a greater proportion of males, higher rates of anxiety, ASD, and learning disorders, and lower rates of depression that the other ED
Eddy et al. (2015)	USA	Cross‐sectional	Retrospective chart review	Patients who presented for an initial evaluation at one of the 19 Boston‐area paediatric gastroenterology clinics affiliated with Massachusetts General Hospital comprising teaching hospital and community settings between January 2008 and December 2008	Paediatric gastroenterology clinics	2231	8–18 years (13.0 ± 3.0; ARFID age = 11.4)	53.4% female	The diagnosis was assigned using a diagnostic checklist based on the proposed DSM‐5 diagnostic criteria	1.5% of participants met ARFID criteria. An additional 2.4% was classified into “possible ARFID” (insufficient information)	67% of ARFID patients were male
Fisher et al. (2015)	USA	Cross‐sectional	Patients referred for an eating disorder evaluation were assigned a DSM‐IV and DSM‐5 diagnosis	Patients who were referred to the division of adolescent medicine for an eating disorders evaluation during the months of September 2011 through December 2012	Outpatient adolescent‐medicine ED programme	309	7–21 years (15.4; ARFID age = 14.6)	83.2% female	DSM‐5 criteria.	19.4% met ARFID criteria during the 16‐month period	ARFID patients were younger and more often male compared to the other EDs. All ARFID were diagnosed with EDNOS with DSM‐IV
Williams et al. (2015)	USA	Cross‐sectional	Chart review	Patients referred to a hospital‐based feeding programme by primary care providers. Many have previously been exposed to treatment and have not been successful in community‐based treatment.	Hospital‐based feeding programme	422	4–219 months (54.5 ± 41.0)	32.0% female	Diagnosis was assigned using three of the four ARFID diagnostic criteria from DSM‐5. The fourth criterion was explicitly omitted (marked interference with psychosocial functioning)	32% met ARFID criteria	
Pinhas et al. (2017)	Australia, Canada, and the UK	Cross‐sectional	Surveillance methodology.Secondary analysis of pooled data. LCA performed on three different data sets	Paediatricians and child psychiatrists were asked to report any child younger than 12 years with a newly diagnosed restrictive ED through three different surveillance units	Children presenting to paediatricians and child and adolescent psychiatrists	436 (352 included in the LCA)	≤12 years (≤13 years in Australia)(Total sample 11.3 ± 1.5; ARFID age = 10.9 ± 1.50)	81.6% female	DSM‐5 criteria.	LCA revealed two distinct clusters. Cluster 2 resembled ARFID and included 25.4% (*n* = 15) of the Australian sample, 29% (*n* = 38) on the Canadian sample, and 34% (*n* = 55) of the UK sample. Cluster 1 resembled AN.	Cluster 2 (congruent with ARFID) was younger (10.9 ± 1.50 vs. 11.6 ± 1.22, *p* < 0.005), more likely to have a co‐morbid psychiatric disorder, specifically an anxiety disorder, and had a longer duration of illness than cluster 1 (congruent with AN)
Cooney et al. (2018)	Canada	Cross‐sectional	Retrospective chart review.	Patients referred for a comprehensive eating disorder assessment in a tertiary care paediatric hospital between May 2013 and April 2016	Paediatric tertiary care ED programme	369	<18 years (ARFID age = 13.2 ± 2.3)		DSM‐5 criteria.	8.4% met ARFID criteria	All the ARFID patients had 2 or more physical symptoms. A co‐morbid psychiatric diagnosis was present in 57.1%
Krom et al. (2019)	The Netherlands	Cross‐sectional	Retrospective chart review. Parents completed questionnaires for additional information	Patients referred by paediatricians or general practitioners because of feeding difficulties to the Diagnostic Centre for feeding problems in the Emma Children's Hospital/Amsterdam UMC (tertiary care) in Amsterdam, The Netherlands, between September 2014 and July 2016	Tertiary care paediatric feeding clinic	100	0–10 years (ARFID age = 1.85)		DSM‐5 criteria.	64% met ARFID criteria	64.1% ARFID patients were female. HRQOL of children with ARFID was lower compared to both healthy and chronically ill controls.
Goldberg et al. (2020)	Canada	Cross‐sectional	Patients who met criteria for the study completed self‐administered questionnaires	All female individuals presenting to the tertiary care Paediatric and adolescent Gynaecology Clinic at the Hospital for Sick Children (SickKids), Toronto, Ontario, Canada, from October 2017 to April 2019	Paediatric and adolescent Gynaecology Clinic	190	8–18 years (ARFID age = 16.3 ± 1.6)	100% female	To identify patients at risk of ARFID, the EDY‐Q was used. This is a self‐report questionnaire based on the DSM‐5 criteria for ARFID, the GOS criteria, and available literature on early‐onset restrictive eating disturbances	3.7% of the sample were at risk for ARFID	43% of patients at risk for ARFID self‐reported anxiety disorders
Schöffel et al. (2021)	Germany	Cross‐sectional	Self‐report questionnaires and medical record review	Patients included were assessed 1–2 days after their admission at the general and neuropaediatric clinic of the University Hospital Leipzig between June 2018 and May 2019	General paediatric inpatient service	111	8–18 years (13.03 ± 2.94; ARFID age = 13.39 ± 2.97)	63.1% female	Symptoms of ARFID were identified using a self‐report questionnaire (EDY‐Q) and medical record review	7.2% of the total sample showed symptoms of ARFID based on self‐report and medical records	10.5% of children and adolescents with GI diseases showed symptoms of ARFID.87.5% of children and adolescents with symptoms of ARFID had underweight.
Farag et al. (2022)	The UK	Cross‐sectional	Data collected prospectively from patients seen by the tertiary feeding service	Paediatric patients experiencing severe feeding difficulties referred to a tertiary feeding clinic at Evelina London Children's Hospital between January 2013 and June 2019	Tertiary care paediatric feeding clinic	536	10 months‐20 years (6years 10 months ± 3years 5 months; ARFID age = 6years 6 months ± 3years)	25.2% female	DSM‐5 criteria.ARFID diagnosis was made by a specialist multidisciplinary team	49.1% of the sample met ARFID criteria	Comorbid ASD was present in 54.75% of patients with ARFID.Younger age, ASD and male sex significantly predicted ARFID in the sample.
Koomar et al. (2021)	USA	Cross‐sectional	Risk for ARFID was estimated for each participant in a large cohort of individuals with ASD	All participants had a diagnosis of ASD and were recruited from a large US‐based multicentric cohort of individuals with ASD and their families (the SPARK project)	Clinical sites across the US participating in the SPARK project.	5157	11.1 ± 5.87	19% female	Individuals at high‐risk for ARFID were identified using the NIAS as well as questions on inflexible eating behaviours and sensory sensitivities, and familial history of ARFID and other eating disorders	21% of probands were at high‐risk for ARFID	17% of parents of children with ASD were at high‐risk for ARFID
Bertrand et al. (2021)	France	Cross‐sectional	Participants were assessed by paediatricians using an anonymised questionnaire	Paediatric patients seeking for consultation from May 2019 to March 2020 in one French department (seine‐maritime, Normandy)	General paediatric population	401	0–18 years		DSM‐5 criteria	The estimated prevalence rate for ARFID was 3%	Only 18% of patients with ARFID were receiving professional care
Katzman et al. (2021)	Canada	Cross‐sectional	National surveillance study: Paediatricians were surveyed monthly and asked to report any new cases that met the criteria for ARFID	Paediatricians were asked to report any child or adolescent aged 5–18 years who was seen in the previous month and met the DSM‐5 diagnostic criteria for ARFID from January 2016 to December 2017 in Canada	Children presenting to paediatricians	2,700^a^	5–18 years (ARFID age = 13.1 ± 3.2)	61.4% female	DSM‐5 criteria	The incidence of ARFID was 2.02 (95% CI, 1.76–2.31) per 100,000 patients	61.4% of ARFID patients were female. 48.8% of ARFID patients had comorbid anxiety. 8.2% of ARFID patients had comorbid ASD.
Nygren et al. (2021)	Sweden	Cross‐sectional	Retrospective chart review	Preschool children born 2010–2016 with ASD from a multi‐ethnic, low resource area in Sweden	Children diagnosed with DSM‐5 ASD by a multidisciplinary team	46	22–59 months at ASD diagnosis (38 months ± 9; ARFID age = 35.2 months)	19.1% female	DSM‐5 criteria (operationalisations were developed).Presence of ARFID was assessed at the time of ASD diagnosis	28% met ARFID criteria	In 69.2% of children with ARFID feeding problems started during the child's first year.38.5% of children with ARFID had ≥1 coexisting medical conditions.
Murray et al. (2022)	USA	Cross‐sectional	Retrospective chart review	Patients who presented for initial evaluation with a neuro‐gastroenterologist in a tertiary care academic medical centre from January 2016 to December 2018	Paediatric tertiary care neuro‐gastroenterology clinic for gastrointestinal functional/motility symptoms	129	6–18 years (ARFID age = 13.9 ± 3.6)	57% female	The diagnosis was assigned using a diagnostic checklist based on the proposed DSM‐5 diagnostic criteria	23% of the sample had ARFID symptoms (8% met full criteria for ARFID and 15% were potential cases)	Patients with ARFID symptoms were older, had lower BMI percentile, and were more likely to be female. The fear of aversive consequences subtype was the most common. The most common GI complaints were constipation and abdominal pain.
Venema et al. (2022)	The Netherlands	Cross‐sectional	Retrospective chart review	Patients with hepatic GSD or IKH seen at the University Medical Centre Groningen between June 2012 and December 2019 who had been referred to SeysCentra	Patients with hepatic GSD or IKH referred to a specialised centre in paediatric feeding and eating issues	16	2.2–23.2 years (6.5; ARFID age = 6.8)	43.8% female	DSM‐5 criteria	31.25% met ARFID criteria	
Wong et al. (2022)	Singapore	Cross‐sectional	Retrospective chart review	Patients diagnosed with EDs and followed up by a specialised paediatric ED management team at a tertiary hospital in Singapore	Paediatric patients with EDs	177	≤18 years (14.6 ± 1.8; ARFID age = 14.7 ± 2.1)	89% female	Not specified. Assessment conducted by a multidisciplinary team including psychiatrists	7% of the sample were ARFID	Half of ARFID patients were male. 41.7% of ARFID patients had ASD.

Abbreviations: AN, anorexia nervosa; ARFID, avoidant/restrictive food intake disorder; ASD, autism spectrum disorder; BMI, body mass index; BN, bulimia nervosa; DSM‐5, Diagnostic and Statistical Manual of Mental Disorders, Fifth Edition; ED, eating disorder; EDNOS, eating disorder not otherwise specified; EDY‐Q, Eating Disturbances in Youth‐Questionnaire; FAED, food avoidant emotional disorder; GOS, Great Ormond Street; GSD, glycogen storage disease; IKH, idiopathic ketotic hypoglycemia; LCA, latent class analysis; NIAS, Nine Item Avoidant/Restrictive Food Intake Disorder Screen; SE, selective eating; SPARK, Simons Foundation Powering Autism Research.

^a^ Surveyed paediatricians.

**TABLE 2:** Included studies using community samples

Author (year)	Country	Design	Methodology	Sample description	Sample setting	Sample size (*n* = )	Sample age	Sample gender	How was the ARFID diagnosis made?	ARFID prevalence/incidence	Other findings
Kurz et al. (2015)	Switzerland	Cross‐sectional	Screening for ARFID in school children using a self‐report questionnaire	Children were recruited from regular schools (3rd–6th grade) in Switzerland in the cities of Fribourg, Lausanne, Bern, and their surrounding areas	Regular schools.	1444	8–13 years (10.55 ± 1.89)	53.9% female	The diagnosis was assigned using a newly constructed self‐report questionnaire, the EDY‐Q based on the DSM‐5 criteria, the GOS criteria, and available literature on early‐onset restrictive eating disturbances	3.2% met ARFID criteria	60.9% of ARFID indicated limited food intake due to the sensory properties of the food; 39.1% lack of interest in eating or food; 15.2% indicated food avoidance based on negative consequences of eating.
Seike et al. (2016a)	Japan	Cross‐sectional	Questionnaire survey asking rates of encounter of ED students. It included an explanation of the DSM‐5 categories and criteria	Participants were yogo teachers^a^ at elementary, junior high, senior high, and special needs schools in Chiba Prefecture, Japan	Yogo teachers	655	6–18 years		DSM‐5 criteria	Encounter rate^b^ for ARFID was 10.7%	ARFID had the highest encounter rate in senior high schools
Seike et al. (2016b)	Japan	Cross‐sectional	Questionnaire survey asking rates of encounter of ED students. It included an explanation of the DSM‐5 categories and criteria	Participants were yogo teachers at elementary, junior high, senior high, and special needs schools in four prefectures in Japan	Yogo teachers	1886	6–18 years		DSM‐5 criteria	Encounter rate^b^ for ARFID was 13.0%	58.8% of yogo teachers reported “do not know well” and 15.4% “do not know anything” about ARFID
Schmidt et al. (2018)	Germany	Cross‐sectional	Data collected from clinical examinations, questionnaires, and interviews. A LCA was performed to delineate subgroups of restrictive eating	Participants from a large prospective population‐based cohort study. Study participants were recruited via advertisement at different institutions	Children and adolescents from the general population	799	7–14 years (10.5 ± 2.02)	46.1% female	The presence of ARFID symptoms was determined using the EDY‐Q, a self‐report questionnaire based on the DSM‐5 criteria for ARFID, the GOS criteria, and available literature on early‐onset restrictive eating disturbances	5.5% of the total sample had ARFID symptoms. 1.4% had ARFID symptoms and objectively measured underweight.	
Gonçalves et al. (2019)	Portugal	Cross‐sectional	Children and their parents completed questionnaires	Children attending primary schools who were fluent in Portuguese language	Public primary schools	330	5–10 years (7.6 ± 1.2)	50.9% female	A parent‐report questionnaire based on the DSM‐5 criteria to assess the presence of ARFID symptoms in their children	15.5% of the sample were possible cases of ARFID	Possible cases of ARFID were significantly associated with anxiety/depression. Possible cases of ARFID were positively and significantly associated with parent's inappropriate eating habits and style and parental pressure to eat.
Chen et al. (2020)	Taiwan	Cross‐sectional	National epidemiological study. Data was collected via questionnaires (participants, parents, and teachers) and psychiatric interviews (participants).	Children from selected grades in 69 schools in Taiwan	Schools	4816	7–14 years	47.7% female	Psychiatric interview using the Mandarin version of the K‐SADS‐E for DSM‐5. Questionnaires were used to obtain relevant information from their parents.	Lifetime prevalence of ARFID was 0.5%. Six‐month prevalence was 0.3%	
Dinkler et al. (2022a)	Japan	Cross‐sectional	Screening for ARFID in the general population using a parent‐report questionnaire	Participants were recruited from a sub‐sample of the JECS project and were children born in the Kochi prefecture between July 2011 and December 2014	Children from the general population	3728	49–95 months (68.1 ± 11; ARFID age = 67.7 ± 12.3)	49.1% female	A parent‐report screening tool based on the DSM‐5 criteria was developed to identify individuals with ARFID, the ARFID‐brief screener (ARFID‐BS).	The prevalence of ARFID was 1.3%.	63% of ARFID indicated limited food intake due to the sensory properties of the food; 51% due to lack of interest in eating; 14% due to fear of aversive consequences of eating.
Iron‐Segev et al. (2022)	Israel	Cross‐sectional	Parents completed questionnaires	Participants were recruited from Jewish orthodox and secular families in Israel	Healthy children from the general population	64	4–12 years	39.06% female	A parent‐report questionnaire to assess children's eating behaviours and parental feeding habits	The prevalence of ARFID was 10.9%	No statistically significant differences in ARFID between religious and non‐religious children
Dinkler et al. (2022b)	Japan	Cross‐sectional	Screening for ARFID in the general population using a parent‐report questionnaire. Calculating a risk score to assess for NDPs between 0.5 and 3 years of age	Participants were recruited from a sub‐sample of the JECS project and were children born in the Kochi prefecture between July 2011 and December 2014	Children of the general population	3728	4–7 (68.1 ± 11; ARFID age = 67.7 ± 12.3)	49.1% female	A parent‐report screening tool based on the DSM‐5 criteria was developed to identify individuals with ARFID, the ARFID‐brief screener (ARFID‐BS)	3.1% of children above the 90^th^ percentile for the NDPs risk score had ARFID	20.8% of children with ARFID scored above the 90^th^ percentile in the NDPs risk score (indicating the presence of 1 or more NDDs) compared to 8.6% of children without ARFID (OR = 2.8 95% CI 1.38–5.67). 8.2% of children with ARFID had a diagnosis of ASD.

Abbreviations: ARFID, avoidant/restrictive food intake disorder; DSM‐5, Diagnostic and Statistical Manual of Mental Disorders, Fifth Edition ED, eating disorder; EDY‐Q, Eating Disturbances in Youth‐Questionnaire; GOS, Great Ormond Street; JECS, Japan Environment and Children's Study; K‐SADS‐E, Kiddie‐Schedule for Affective Disorders and Schizophrenia ‐ Epidemiological version; LCA, latent class analysis; NDD, Neurodevelopmental disorder; NDP, Neurodevelopmental problem.

^a^ Yogo teachers are particular type of teachers in Japan that monitor students' height and weight and are in charge of health education.

^b^ Encounter rate = proportion of Yogo teachers who had encountered students with ARFID (calculated by dividing the number of Yogo teachers who had encountered ARFID by the total number of Yogo teachers who submitted the survey. Note that the encounter rate is not a prevalence rate, but the proportion of Yogo teachers who had encounter ARFID.

### 3.2 Quality assessment

The AXIS tool (Downes et al., 2016) was used to assess the quality of each study and this is detailed in Table 3. The total score for the different studies ranged from 8 to 20 with one achieving a maximum score of 20 (Chen et al., 2020) and only two having a score of less than 10 (Fisher et al., 2015; Norris et al., 2014). All the included studies had clear objectives, used an appropriate study design, and had a clearly defined target population.

**TABLE 3:** Quality assessment of the included studies using the AXIS tool

	Clear objectives	Appropriate study design	Sample size justified	Population defined	Sample appropriately taken	Sample selection	Addressing non‐responders correctly	Measurement of outcome variables	Correct measurement of outcome variables	Statistical significance measured clearly	Correct methodology details	Description of basic data	Non‐response bias addressed	Information on non‐responders described	Were the results internally consistent	Analysis and methodology matched	Results and discussion matched	Addressing limitations	Funding or COI declared	Ethical approval and patient consent	Total score
Ornstein et al. (2013)	Yes	Yes	No	Yes	Yes	Yes	NK	Yes	No	No	No	Yes	NK	NK	No	Yes	Yes	Yes	Yes	Yes	12
Fisher et al. (2014)	Yes	Yes	No	Yes	Yes	No	NK	Yes	No	No	Yes	Yes	NK	NK	Yes	Yes	Yes	Yes	Yes	Yes	13
Norris et al. (2014)	Yes	Yes	No	Yes	No	No	NK	Yes	No	No	No	Yes	NK	NK	No	Yes	Yes	Yes	No	No	8
Forman et al. (2014)	Yes	Yes	No	Yes	Yes	No	NK	Yes	No	Yes	Yes	Yes	Yes	No	Yes	Yes	Yes	Yes	Yes	Yes	15
Nicely et al. (2014)	Yes	Yes	No	Yes	Yes	No	NK	Yes	No	Yes	Yes	Yes	NK	NK	Yes	Yes	Yes	Yes	Yes	Yes	14
Eddy et al. (2015)	Yes	Yes	No	Yes	Yes	No	NK	Yes	Yes	No	Yes	Yes	NK	NK	Yes	Yes	Yes	Yes	No	No	12
Kurz et al. (2015)	Yes	Yes	No	Yes	Yes	Yes	NK	Yes	Yes	Yes	Yes	Yes	Yes	Yes	Yes	Yes	Yes	Yes	Yes	Yes	18
Fisher et al. (2015)	Yes	Yes	No	Yes	Yes	No	NK	Yes	No	No	No	Yes	NK	NK	Yes	Yes	Yes	No	No	No	9
Williams et al. (2015)	Yes	Yes	No	Yes	Yes	No	NK	Yes	No	No	No	Yes	NK	NK	Yes	Yes	Yes	Yes	No	Yes	11
Seike et al. (2016a)	Yes	Yes	Yes	Yes	No	No	No	No	No	No	Yes	Yes	No	No	Yes	Yes	Yes	Yes	Yes	Yes	12
Seike et al. (2016b)	Yes	Yes	Yes	Yes	Yes	No	No	No	No	No	Yes	Yes	No	No	No	Yes	Yes	Yes	Yes	Yes	12
Pinhas et al. (2017)	Yes	Yes	No	Yes	Yes	No	Yes	Yes	No	Yes	Yes	Yes	Yes	Yes	Yes	Yes	Yes	Yes	Yes	Yes	17
Cooney et al. (2018)	Yes	Yes	No	Yes	Yes	No	No	Yes	No	No	Yes	Yes	Yes	No	Yes	Yes	Yes	Yes	Yes	Yes	14
Schmidt et al. (2018)	Yes	Yes	Yes	Yes	Yes	Yes	Yes	Yes	No	Yes	Yes	Yes	Yes	Yes	Yes	Yes	Yes	Yes	Yes	Yes	19
Gonçalves et al. (2019)	Yes	Yes	No	Yes	No	No	NK	Yes	No	Yes	Yes	Yes	NK	NK	Yes	Yes	Yes	Yes	Yes	Yes	13
Chen et al. (2019)	Yes	Yes	Yes	Yes	Yes	Yes	Yes	Yes	Yes	Yes	Yes	Yes	Yes	Yes	Yes	Yes	Yes	Yes	Yes	Yes	20
Krom et al. (2019)	Yes	Yes	Yes	Yes	Yes	No	Yes	Yes	No	Yes	Yes	Yes	Yes	Yes	Yes	Yes	Yes	Yes	No	Yes	17
Goldberg et al. (2020)	Yes	Yes	No	Yes	Yes	Yes	NK	Yes	No	No	No	Yes	NK	NK	Yes	Yes	Yes	Yes	Yes	Yes	13
Schöffel et al. (2021)	Yes	Yes	Yes	Yes	Yes	No	Yes	Yes	Yes	Yes	Yes	Yes	Yes	Yes	Yes	Yes	Yes	Yes	Yes	Yes	19
Farag et al. (2022)	Yes	Yes	No	Yes	Yes	No	NK	Yes	No	Yes	Yes	Yes	NK	NK	No	Yes	Yes	Yes	Yes	Yes	13
Koomar et al. (2021)	Yes	Yes	No	Yes	Yes	Yes	No	Yes	No	Yes	Yes	Yes	No	No	Yes	Yes	Yes	Yes	Yes	Yes	15
Bertrand et al. (2021)	Yes	Yes	No	Yes	Yes	Yes	NK	Yes	No	Yes	Yes	Yes	Yes	NK	Yes	Yes	Yes	Yes	Yes	Yes	16
Dinkler and Bryant‐Waugh (2021)	Yes	Yes	No	Yes	Yes	Yes	Yes	Yes	No	Yes	Yes	Yes	Yes	Yes	Yes	Yes	Yes	Yes	Yes	Yes	18
Katzman et al. (2021)	Yes	Yes	No	Yes	Yes	Yes	No	Yes	No	Yes	Yes	Yes	Yes	NK	Yes	Yes	Yes	Yes	Yes	Yes	16
Nygren et al. (2021)	Yes	Yes	No	Yes	Yes	No	No	Yes	Yes	Yes	Yes	Yes	NK	NK	Yes	Yes	Yes	No	Yes	Yes	14
Murray et al. (2022)	Yes	Yes	No	Yes	Yes	No	No	Yes	No	Yes	Yes	Yes	NK	NK	Yes	Yes	Yes	Yes	Yes	Yes	14
Venema et al. (2022)	Yes	Yes	No	Yes	No	No	No	Yes	No	Yes	Yes	Yes	NK	NK	Yes	Yes	Yes	Yes	Yes	Yes	13
Iron‐Segev et al. (2022)	Yes	Yes	Yes	Yes	No	No	NK	Yes	No	Yes	Yes	Yes	NK	NK	Yes	Yes	Yes	Yes	Yes	Yes	14
Dinkler et al. (2022)	Yes	Yes	No	Yes	Yes	Yes	Yes	Yes	No	Yes	Yes	Yes	Yes	Yes	Yes	Yes	Yes	Yes	Yes	Yes	18
Wong et al. (2022)	Yes	Yes	No	Yes	Yes	No	No	No	No	Yes	Yes	Yes	NK	NK	Yes	Yes	Yes	Yes	Yes	Yes	13

Abbreviation: NK, not known.

### 3.3 Results from the included studies

**FIGURE 2:**
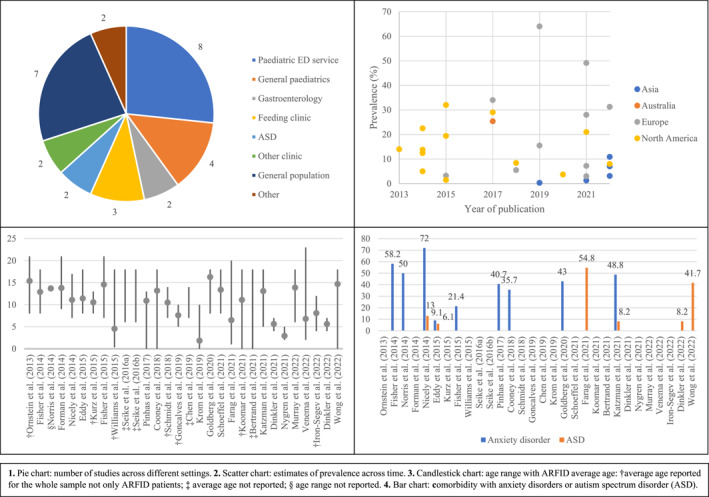
Summary of results

A summary of the results is presented in Figure 2. A significant proportion of the existing literature on the epidemiology of ARFID comes from specialised paediatric eating disorders treatment settings (8 of 29 studies), where prevalence rates of ARFID ranged from 5% to 22.5% (Cooney et al., 2018; Fisher et al., 2014, 2015; Forman et al., 2014; Nicely et al., 2014; Norris et al., 2014; Ornstein et al., 2013; Wong et al., 2022). Most of these clinic‐based studies used retrospective reviews of clinical records to ascertain the presence or absence of ARFID symptoms (7 of 8 studies). Three studies were conducted in specialised tertiary care services for feeding problems and they showed the highest prevalence rates of ARFID, ranging from 32% to 64% (Farag et al., 2022; Krom et al., 2019; Williams et al., 2015). Two studies examined the prevalence of ARFID in general paediatric services, with a prevalence of 3% in an outpatient sample (Bertrand et al., 2021) and of 7.2% in an inpatient sample (Schöffel et al., 2021). Two studies took place in paediatric gastroenterology clinics and reported prevalence estimates ranging from 1.5% to 8% with an additional 2.4%–15% of possible/potential cases (i.e., when subjects met some criteria for ARFID, but not enough information was available to make a full diagnosis) (Eddy et al., 2015; Murray et al., 2022). Furthermore, one study reported a prevalence of 31.25% in a small sample of children and adolescents with congenital metabolic disorders (Venema et al., 2022). One study was conducted in a paediatric and adolescent gynaecology clinic, where a prevalence of ARFID of 3.7% was found (Goldberg et al., 2020). One of the studies included in this review used surveillance methodology and found the incidence of ARFID in children and adolescents aged 5–18 in Canada was 2.02 (95%CI, 1.76–2.31) per 100,000 patients (Katzman et al., 2021). Finally, studies from non‐clinical samples used self‐report or parent‐report instruments (6 of 29 studies) with ARFID estimates ranging from 0.3% to 15.5% (Chen et al., 2020; Dinkler et al., 2022a; Gonçalves et al., 2019; Iron‐Segev et al., 2022; Kurz et al., 2015; Schmidt et al., 2018) and one used a risk score to identify ARFID in children with neurodevelopmental problems (those on the 90^th^ percentile for neurodevelopmental problems presented with a risk of 3.1% of having ARFID) (Dinkler et al., 2022b). In addition, two other studies reported that the encounter rate of students with ARFID by *Yogo* teachers (a particular type of teachers in Japan responsible for monitoring students' height and weight and leading on health education) ranged from 10.7% to 13% (Seike et al., 2016a, 2016b). The encounter rate is not a prevalence rate, but rather the proportion of *Yogo* teachers who had encountered students with ARFID (one or more).

Although ARFID captures a range of clinical presentations, as data from these studies show, children and adolescents with this disorder are clinically different from those with AN and BN. Studies conducted in eating disorders services found that patients with ARFID are generally younger at presentation: the mean age in ARFID ranged from 11.1 to 14.6 years versus 14–15.6 years in AN and 14.9–16.7 years in BN (Fisher et al., 2014, 2015; Forman et al., 2014; Nicely et al., 2014; Norris et al., 2014). The diagnosis tends to be more common in males (proportion of males ranging from 21% to 50%) (Cooney et al., 2018; Fisher et al., 2014, 2015; Forman et al., 2014; Nicely et al., 2014; Norris et al., 2014; Wong et al., 2022) and have a longer duration of illness prior to diagnosis compared to other EDs (12–33 months in ARFID vs. 8–23 months in other EDs) (Fisher et al., 2014; Forman et al., 2014). Many of these studies were conducted in specialised eating disorders services where females were overrepresented, which may account for the comparatively lower proportion of males with ARFID in some of these studies (Cooney et al., 2018; Fisher et al., 2014, 2015; Forman et al., 2014; Nicely et al., 2014; Norris et al., 2014) than in general population samples and a general paediatric sample, which reported that ARFID is equally common in boys and girls (Dinkler et al., 2022a; Gonçalves et al., 2019; Kurz et al., 2015; Schöffel et al., 2021).

Young people with ARFID often present with a comorbid psychiatric disorder or medical conditions/symptoms. Anxiety disorders were most common, with estimates ranging from 9.1% to 72% (Cooney et al., 2018; Eddy et al., 2015; Fisher et al., 2014, 2015; Goldberg et al., 2020; Katzman et al., 2021; Nicely et al., 2014; Norris et al., 2014; Pinhas et al., 2017) and generalised anxiety disorder was most frequently reported, with estimates from 21.4% to 50% (Fisher et al., 2014, 2015; Norris et al., 2014). In contrast, comorbid depressive illness was less frequent (7.2%–33%) than for young people with AN (19.4%–48%) or BN (23.1%–80%) (Fisher et al., 2014; Nicely et al., 2014). Other common comorbidities with ARFID are neurodevelopmental disorders, especially ASD (Farag et al., 2022). This is supported by findings from this review, with 21%–28% of children with ASD at high‐risk of ARFID (Koomar et al., 2021; Nygren et al., 2021) and the prevalence of ASD among children with ARFID ranging from 8.2% to 54.75% (Dinkler et al., 2022b; Eddy et al., 2015; Farag et al., 2022; Katzman et al., 2021; Nicely et al., 2014; Wong et al., 2022). In terms of medical comorbidity, two studies reported that 45%–51% of patients with ARFID had medical symptoms (Fisher et al., 2014; Katzman et al., 2021) and 6 studies were conducted in clinical contexts where all patients presented with physical or medical symptoms (Bertrand et al., 2021; Eddy et al., 2015; Goldberg et al., 2020; Murray et al., 2022; Schöffel et al., 2021; Venema et al., 2022). Comorbidity with gastrointestinal (GI) symptoms or disorders was reported in 19.4%–43.8% of patients with ARFID (Fisher et al., 2014, 2015; Krom et al., 2019).

There is a lack of clarity with regard to differences in ARFID prevalence according to age. Study populations vary widely and include different age ranges, so it is hard to make comparisons between studies. However, reports from the Japanese survey of *Yogo* teachers in Japan suggested a similar encounter rate for ARFID in elementary, junior, and senior school pupils (Seike et al., 2016b) while a French study of paediatric patients aged 0–18 years found the highest prevalence in the 1–6 year old group (Bertrand et al., 2021). Similarly, a UK study conducted in a tertiary feeding clinic that included patients younger than 1 year up to 20 years old reported that ARFID was more prevalent in the 4–9 years group than in the other groups. By contrast, the Canadian national surveillance study of ARFID with patients aged 5–18 years found the highest incidence in the 10–14 years old group (3.43 per 100,000 patients) (Katzman et al., 2021). The average age of patients with ARFID in the included studies ranged from 1.85 to 16.3 years (Cooney et al., 2018; Dinkler et al., 2022a, 2022b; Eddy et al., 2015; Farag et al., 2022; Fisher et al., 2014, 2015; Forman et al., 2014; Goldberg et al., 2020; Katzman et al., 2021; Krom et al., 2019; Murray et al., 2022; Nicely et al., 2014; Norris et al., 2014; Nygren et al., 2021; Pinhas et al., 2017; Schöffel et al., 2021; Venema et al., 2022; Wong et al., 2022).

## 4 DISCUSSION

To our knowledge, this is the first systematic review to exclusively focus on the epidemiology of ARFID in children and adolescents. Knowledge about the epidemiology of a disorder is fundamental in order to understand the needs for clinical service provision and to identify populations at risk. The studies that were included in this review were very heterogeneous and came from different settings, used different methodologies, and had small samples. Different clinical presentations that were seen in different settings were diagnosed as ARFID, with estimates of prevalence fluctuating across studies. The wide variation in estimates complicates the development of adequate care pathways for this patient group. Some of the clinical settings were specific services such as a paediatric and adolescent gynaecology clinic (Goldberg et al., 2020) or a centre for metabolism diseases (Venema et al., 2022) which makes it difficult to extrapolate these results to other settings. The highest prevalence estimates in clinical settings were reported in paediatric feeding clinics with up to 64% of individuals meeting criteria for ARFID (Farag et al., 2022; Krom et al., 2019; Williams et al., 2015). Patients referred to these services have more severe feeding difficulties and have previously been exposed to unsuccessful treatments in other settings with ARFID likely unrecognised. Results from studies that retrospectively reviewed medical records in paediatric eating disorders services showed that less than a quarter of the patients in these settings were diagnosed with ARFID (prevalence estimates ranging from 5% to 22.5%) (Cooney et al., 2018; Fisher et al., 2014, 2015; Forman et al., 2014; Nicely et al., 2014; Norris et al., 2014; Ornstein et al., 2013; Wong et al., 2022). The majority of these studies were conducted in North America and studies from other geographical areas are needed to make comparisons. Estimates in other clinical settings were lower but still substantial, with prevalence rates ranging from 3% to 7.2% in general paediatric services (Bertrand et al., 2021; Schöffel et al., 2021) and from 1.5% to 8% in paediatric gastroenterology clinics (Eddy et al., 2015; Murray et al., 2022). It is important that clinicians are aware of ARFID and systems to identify it and produce appropriate referrals are developed, especially in feeding and eating disorders clinics and in services working with children and young people with medical problems.

Only one study reported incidence, which is necessary for anticipating clinical demand for care. The study conducted in Canada reported that the incidence of ARFID was 2.02 per 100,000 young people aged 5–18 presenting to paediatricians which suggests that new presentations to clinical care are relatively rare (Katzman et al., 2021). This is the first to use active national surveillance to study ARFID and to look at the incidence. This study was conducted in collaboration with the Canadian Paediatric Surveillance Program. Paediatric surveillance units (PSUs) such as this are currently established in 12 countries in order to study rare paediatric diseases. In 2007, this was supplemented in Britain with the launch of the first mental health specific surveillance system, the Child and Adolescent Psychiatric Surveillance System (CAPSS). PSUs facilitate study investigators to undertake national, prospective, timely and active surveillance for uncommon paediatric disorders and this methodology has proved to be very useful in studying the epidemiology of EDs in children and adolescents (Katzman et al., 2017). Further studies using surveillance methodology in different countries should be conducted in order to better understand the epidemiology and clinical characteristics of ARFID in children and young people reaching paediatric or psychiatric care. This methodology also allowed ascertainment of data from a large, representative, community‐based sample with children and adolescents from different ages across a variety of geographical areas increasing understanding of the clinical and demographic characteristics of the disorder. Whilst ARFID diagnoses were made by the reporting paediatricians, the research team looked at each case to confirm that they met the DSM‐5 criteria for ARFID and could be included in the study, ensuring strong diagnostic reliability. Additionally, the use of the highly rigorous protocol from the Canadian Paediatric Surveillance Program in this study increased generalisability and will allow comparison of the results with those from PSUs in other countries.

ARFID is still often unrecognised by health professionals. A small survey of clinicians in Canada reported that less than 50% of the participants had heard of ARFID previously (Magel et al., 2021). Similarly, a survey of *Yogo* teachers (responsible for monitoring students' height and weight and leading on health education), found that only 13% of the participants had encountered students with symptoms of ARFID (Seike et al., 2016b). When asked further about their knowledge of ARFID, almost 60% indicated a lack of experience with this diagnostic group, with a further 15% indicating a complete lack of familiarity (Seike et al., 2016b). Another survey of multi‐disciplinary health professionals working across a range of treatment settings, showed that knowledge about this disorder has improved in recent years, with 78.5% of participants reporting familiarity with ARFID. However, participants reported a lack of confidence in treating this group of patients (Coelho et al., 2021), with a recent study from France reporting that only 18% of children with ARFID in their sample were receiving professional care (Bertrand et al., 2021). Following the publication of DSM‐5, the diagnostic specificity of eating disorders in young people has improved. Studies that reviewed the medical records of patients with a DSM‐IV eating disorder diagnosis showed that all patients with a DSM‐5 diagnosis of ARFID were classified as eating disorder not otherwise specified (EDNOS) in DSM‐IV (Fisher et al., 2015; Nicely et al., 2014; Ornstein et al., 2013).

Diagnosing ARFID can be complex and challenging. It may present with a variety of different physical symptoms with referrals to a range of specialities (Eddy et al., 2015; Goldberg et al., 2020). Many patients diagnosed with ARFID seek clinical attention due to complaints of low weight, weight loss, or failure to thrive (Eddy et al., 2015). Common associated physical symptoms are gastroesophageal reflux, constipation, abdominal pain, vomiting or early satiety. On many occasions these physical symptoms predate the eating disturbance, with almost 50% of patients with ARFID having consultations with different disciplines such as gastroenterology or endocrinology prior to referral to more specialist services (Cooney et al., 2018; Nicely et al., 2014). This suggests that the recognition of ARFID is difficult for health care providers and the complexity of these cases often requires a comprehensive physical assessment and further investigations to exclude underlying medical conditions.

The results from this review show that young people with ARFID have high rates of comorbidity. This is consistent with one study of 74 children and adolescents with ARFID of whom 45% met criteria for a current psychiatric comorbidity and 53% for a lifetime comorbid diagnosis (Kambanis et al., 2020). The most common comorbidity was anxiety, which can be as high as 72% (Nicely et al., 2014). Therefore, treatment for anxiety disorders should be included in the models of care for ARFID. Neurodevelopmental disorders are also common, especially ASD. Results from this review showed that the prevalence of ARFID in individuals with ASD can be as high as 28% (Koomar et al., 2021; Nygren et al., 2021) and children with several neurodevelopmental problems had three times higher odds of having ARFID (Dinkler et al., 2022b). This is consistent with a meta‐analysis which found that feeding difficulties were five times more frequent in children with ASD than in typically developing children, with higher levels of nutritional deficiency in the ASD population (Sharp et al., 2013). It seems that, in a proportion of cases, feeding difficulties in association with ASD are in fact likely to meet criteria for ARFID. Sensory sensitivities overlap in both disorders and there may be shared underlying aetiological mechanisms. ARFID assessment and management should be included in the care pathways of children with ASD. Furthermore, adaptations to the methods of delivery of interventions for young people with autism and ARFID should be developed and implemented (Bryant‐Waugh et al., 2021).

This review found that 19%–44% of patients with ARFID have gastrointestinal (GI) symptoms or disorders (Fisher et al., 2014, 2015; Krom et al., 2019). The study that examined the prevalence of ARFID in a general paediatric inpatient reported a higher estimate in those patients that were admitted for a GI problem (10.5 vs. 7.2%, not significantly different) (Schöffel et al., 2021) and the prevalence of ARFID can be as high as 23% in paediatric gastroenterology clinics (Eddy et al., 2015; Murray et al., 2022). In addition to these results, one study from 2020 reported that over 60% of children with ARFID admitted to a paediatric hospital had some past history of GI problems (Tsang et al., 2020). These estimates, although different from each other, suggest that there might be an overlap between ARFID and GI problems, which could be important for understanding the aetiology of some presentations (Nicholas et al., 2021). The presence of GI symptoms together with ARFID should inform case formulation with assessment and intervention for GI symptoms being a clinical priority in this patient group (Boerner et al., 2021; Nicholas et al., 2021).

The overlap between ARFID and other psychiatric or medical disorders has been acknowledged with some ongoing uncertainty about the validity of ARFID as a specific diagnosis (Strand et al., 2019). It has been argued by some that in view of this, the current diagnostic criteria for ARFID may not be optimal for clinical practice and epidemiological research (Strand et al., 2019) and so, the findings of this review need to be interpreted with this in mind. Further research is likely to be needed to improve the conceptual validity of ARFID (Strand et al., 2019).

Studies using general population samples also showed a wide range of prevalence estimates, from 0.3% to 15.5% (Chen et al., 2020; Dinkler et al., 2022a; Gonçalves et al., 2019; Iron‐Segev et al., 2022; Kurz et al., 2015; Schmidt et al., 2018). These studies were conducted in countries from Europe and Asia and used different diagnostic instruments to assess for the presence of ARFID. This highlights the importance of a robust assessment tool for ARFID when studying its epidemiology (Bourne et al., 2020). The Eating Disturbances in Youth‐Questionnaire (EDY‐Q), a self‐report screening instrument based on the DSM‐5 and Great Ormond Street criteria (Bryant‐Waugh & Lask, 1995), that has shown adequate discriminant, divergent, and convergent validity in general population samples, was used by some studies (Goldberg et al., 2020; Kurz et al., 2015; Schmidt et al., 2018; Schöffel et al., 2021). Using this questionnaire one study reported that 3.2% of children in Switzerland were at risk for ARFID (Kurz et al., 2015). The ARFID‐Brief Screener (ARFID‐BS), a parent‐report questionnaire for children aged 4–7 years, was used in another study and showed satisfactory convergent validity in a Japanese sample with 1.3% at risk for ARFID (Dinkler et al., 2022a). However, neither of these two screening instruments has been validated against clinical diagnoses of ARFID, which likely contributed to the variety of prevalence estimates (Dinkler & Bryant‐Waugh, 2021). The Nine‐Item ARFID Screen (NIAS) has been validated in both general and clinical samples (Dinkler & Bryant‐Waugh, 2021) and was used in one study (Koomar et al., 2021). The screening tools are generally designed towards high sensitivity not to miss potential cases which increases the risk for false positives. The lowest prevalence estimates (0.3%–0.5%) were reported in a study with Taiwanese schools. They used the Kiddie Schedule for Affective Disorders and Schizophrenia‐Epidemiological version (K‐SADS‐E) which is a diagnostic interview and should have a higher specificity (true negatives) than the screening tools (Chen et al., 2020). The two studies that reported the highest prevalence in community samples (i.e., 10.9% and 15.5%) used instruments that did not include items assessing the DSM‐5 exclusion criteria in the parent questionnaires. These studies also stated that the importance of food and eating in a particular culture may have influenced parent‐reports, taking into account cross‐cultural factors (Gonçalves et al., 2019; Iron‐Segev et al., 2022). Further instruments are being developed and among the most promising is the Pica, ARFID, and Rumination Disorder Interview (PARDI) which is a multi‐format, semi‐structured interview that has shown evidence of reliability and validity in diagnosing ARFID (Bryant‐Waugh et al., 2019). However, this may be too costly and time consuming to administer for large scale epidemiological studies and may be better utilised in two stage designs to validate the diagnosis in those identified using screening tools. The use of standardised and well‐validated instruments administered by trained individuals in future studies will likely help to clarify the epidemiology of ARFID. Another challenge to be considered is the need for clearer guidelines in operationalising the DSM‐5 diagnostic criteria for ARFID, with a recent study indicating that prevalence estimates can vary widely depending on whether a strict or broad definition of ARFID is utilised (Harshman et al., 2021). Finally, the instruments should be validated in different countries, age ranges, in males and females, in neurotypical and neurodiverse individuals, and in those with medical or psychiatric comorbidities (Dinkler & Bryant‐Waugh, 2021).

### 4.1 Limitations

There are some limitations of this review that may impact the ability to make firm conclusions about the epidemiology of ARFID. The search was limited to three data bases and conference abstracts were not included, which might have neglected relevant information. Only studies published in the English language were examined, which may have led to omission of significant articles. Studies focussing on the epidemiology of ARFID in adults were not included as this review aimed to explore the epidemiology of ARFID in children and adolescents which led to the exclusion of some important research. Five of the included studies (Farag et al., 2022; Fisher et al., 2015; Forman et al., 2014; Ornstein et al., 2013; Venema et al., 2022) had samples with an age range slightly above 18 years. These were conducted in paediatric or adolescent clinics that also treat young adults and reported mean ages below 18 years, so were included. The included studies were very heterogeneous. Especially, two papers reported the encounter rate of ARFID by *Yogo* teachers (Seike et al., 2016a, 2016b) which is a very different estimate from the ones reported in other studies but these two provided important information about the impact of this disorder in schools. Finally, the current ARFID DSM‐5 diagnostic criteria may not ensure optimal diagnostic validity to be useful in epidemiological research (Strand et al., 2019). The results need to be interpreted in the context of these limitations.

## 5 CONCLUSION

The current literature on the epidemiology of ARFID in children and adolescents is limited. The studies on this topic are heterogeneous with regard to settings and methodologies with a wide range of estimates for prevalence and incidence. Studies on the epidemiology of ARFID provide valuable information but were limited in sample size, scope, setting and generalisability. Although ARFID includes different clinical presentations, its demographic characteristics differ from those of other EDs. Future research is needed to clarify the frequency of this disorder in clinical and community populations and surveillance methodology may have particular advantages in this respect.

## DISCLAIMER

This article presents independent research commissioned by the NIHR under the ARC programme for Northwest London. The views expressed in this publication are those of the author(s) and not necessarily those of the National Health Service (NHS), the NIHR or the Department of Health.

## Data Availability

The data that support the findings of this study are available from the corresponding author upon reasonable request.
